# Association between Epstein–Barr viral load and duration of hospitalization in pediatric infectious mononucleosis: a single-center retrospective study

**DOI:** 10.3389/fmed.2026.1788859

**Published:** 2026-03-20

**Authors:** Zhi Yang, Zongtao Ma, Qingfeng Fang, Biquan Chen, Yuan Xu

**Affiliations:** 1 Department of Pediatric Infectious Disease, Anhui Provincial Children’s Hospital, Hefei, China; 2 Department of Pediatrics, Lixin Hospital of Chinese Medicine, Bozhou, China; 3 Department of Pediatric Nephrology, Anhui Provincial Children’s Hospital, Hefei, China

**Keywords:** children, clinical outcome, Cox regression analysis, Epstein–Barr virus, length of stay, viral load

## Abstract

**Background:**

Epstein–Barr virus (EBV) infection exhibits considerable heterogeneity in clinical manifestations among children, ranging from self-limiting symptoms to severe complications. The role of viral load in disease progression remains incompletely understood.

**Objective:**

To investigate the relationship between EBV DNA load levels and clinical characteristics, treatment response, and prognosis in pediatric patients.

**Methods:**

A total of 192 children diagnosed with EBV infection and admitted to our hospital between January 2022 and October 2025 were included in the final analysis. Based on plasma EBV DNA levels at admission, patients were stratified into a high viral load group (*n* = 99) and a low viral load group (*n* = 93). Baseline clinical data, treatment regimens, virological and serological dynamics, incidence of complications, and length of hospital stay were collected and compared between the two groups. A multivariate Cox proportional hazards regression model was employed to identify independent factors influencing hospitalization duration.

**Results:**

Comparative analysis of baseline characteristics revealed that the high viral load group exhibited significantly elevated peak alanine aminotransferase (ALT) (*p* < 0.001), peak aspartate aminotransferase (AST) (*p* < 0.001), and proportion of atypical lymphocytes (*p* = 0.036) relative to the low viral load group. Regarding treatment, glucocorticoid administration was more frequent in the high viral load group (*p* = 0.002), and the duration of hospitalization was prolonged (*p* < 0.001). Virologically, EBV DNA levels were higher in the high viral load group both at admission (all *p* < 0.001), with significant reduction observed in both groups by discharge (all *p* < 0.001). The incidence of overall complications (*p* < 0.001) and splenomegaly (*p* = 0.001) was greater in the high viral load group. Multivariate Cox regression analysis, with hospitalization duration as the dependent variable, identified high viral load (HR = 0.528, *p* < 0.001) and splenomegaly (HR = 0.665, *p* = 0.016) as independent predictors of prolonged hospital stay.

**Conclusion:**

High EBV DNA load is independently associated with more severe hepatic injury, increased risk of complications, greater need for immunomodulatory therapy, and extended hospitalization. Viral load monitoring may facilitate early identification of children at high risk of unfavorable clinical outcomes.

## 1 Introduction

Epstein–Barr virus (EBV), a ubiquitous human herpesvirus, exhibits high seroprevalence worldwide and represents a significant public health burden, particularly in the pediatric population (1). Primary infection commonly occurs during childhood and demonstrates marked clinical heterogeneity, ranging from subclinical manifestations to classic infectious mononucleosis. The pathophysiological process involves direct viral infection of B lymphocytes and subsequent triggering of a complex cellular immune response (2). The intensity and scope of this immune reaction largely determine the clinical spectrum of the disease, which may vary from self-limited fever and pharyngitis to severe complications such as hepatic and splenic injury, hematological abnormalities, and life-threatening hemophagocytic lymphohistiocytosis (3). Consequently, early identification of children at risk of severe disease progression and implementation of precise risk-stratified management constitute urgent challenges in modern pediatric infectology (4).

Within the clinical evaluation framework for EBV infection, quantitative viral load assessment has demonstrated considerable utility (5). EBV DNA levels in plasma or peripheral blood mononuclear cells provide a direct reflection of viral replication activity and are theoretically closely associated with both direct viral pathogenicity and immune activation burden (6). Previous studies involving post-transplant lymphoproliferative disorders and immunocompromised populations have established high viral load as a key biomarker for predicting adverse outcomes and guiding preemptive antiviral therapy (7). However, the clinical interpretation of viral load is more complex in the context of community-acquired infection among immunocompetent children (8). Its significance extends beyond mere quantification of viral replication, potentially serving as an integrative indicator reflecting the dynamic interplay between the virus and host immune system, which directly influences tissue damage extent and disease trajectory (9). Despite its acknowledged potential importance, the evidence base for systematically incorporating viral load into routine clinical decision-making pathways for pediatric EBV infection remains limited, with a scarcity of large-scale, prospective studies to precisely quantify its association with multidimensional clinical outcomes (10).

Current research in this field exhibits several methodological limitations. First, the majority of studies focus on adult or special immunocompromised populations, raising concerns regarding the validity of extrapolating these findings to children, whose immune systems undergo continuous development and maturation (11). Pediatric immune response characteristics, viral clearance kinetics, and tissue repair capacities differ substantially from those of adults, necessitating dedicated investigations in child-specific cohorts. Second, most existing studies employ cross-sectional designs, providing only static snapshots of associations at single time points, thereby unable to elucidate causal or temporal relationships between dynamic viral load evolution and clinical disease progression (12). For instance, whether early viral load trends possess greater predictive value than single baseline measurements remains unknown. Third, outcome assessment in previous research has often focused on single-organ damage or specific complications, lacking a comprehensive, integrated prognostic evaluation system (13). The systemic nature of EBV infection requires multidimensional assessment encompassing hepatic injury, hematological involvement, treatment intensity requirements (e.g., glucocorticoid use), complication incidence, and healthcare resource utilization (e.g., length of hospitalization). Fourth, technical standardization challenges exist in detection methodologies, including variations in nucleic acid extraction protocols, amplification target selection, and quantitative calibrators, which may hinder direct comparison of results across studies and impede evidence accumulation (14). Finally, and most critically, few studies have employed advanced multivariate statistical models to clarify whether viral load constitutes an independent determinant of prognosis after controlling for potential confounding factors such as age, baseline immune status, and co-infections (15). These methodological gaps hinder the translation of viral load from a laboratory parameter into a reliable clinical tool.

To address these knowledge deficits, we conducted a retrospective cohort study. Immunocompetent children with EBV infection were identified from electronic medical records, and their data were analyzed using validated, standardized real-time quantitative PCR results to assess dynamic viral load changes from the acute phase through convalescence. A key innovation involves constructing a multidimensional clinical outcome assessment framework incorporating traditional biochemical markers (e.g., liver enzymes), immune activation markers (atypical lymphocytes), treatment intervention intensity (glucocorticoid use), typical complications (e.g., splenomegaly), and objective healthcare outcomes (length of hospitalization). Beyond descriptively comparing clinical characteristics across viral load strata, this study will utilize advanced statistical approaches, including multivariate Cox proportional hazards regression, to isolate confounding effects and determine the independent contribution and weight of viral load in predicting adverse clinical outcomes. Our ultimate objective is to provide robust data and a theoretical foundation for establishing an evidence-based, viral load-informed risk stratification and management pathway for pediatric EBV infection, thereby advancing clinical practice toward more precise and individualized care.

## 2 Materials and methods

This study aimed to investigate the impact of initial Epstein–Barr virus (EBV) DNA load on the clinical course and outcomes of children with infectious mononucleosis (IM). Designed as a single-center, retrospective cohort study, it adhered strictly to the reporting standards for observational studies in clinical epidemiology. All procedures complied with the principles of the Declaration of Helsinki and received approval from the institutional ethics review board (Approval no.: EYLL-2025-106). All data were anonymized to ensure patient privacy.

### 2.1 General information

**Figure 1:**
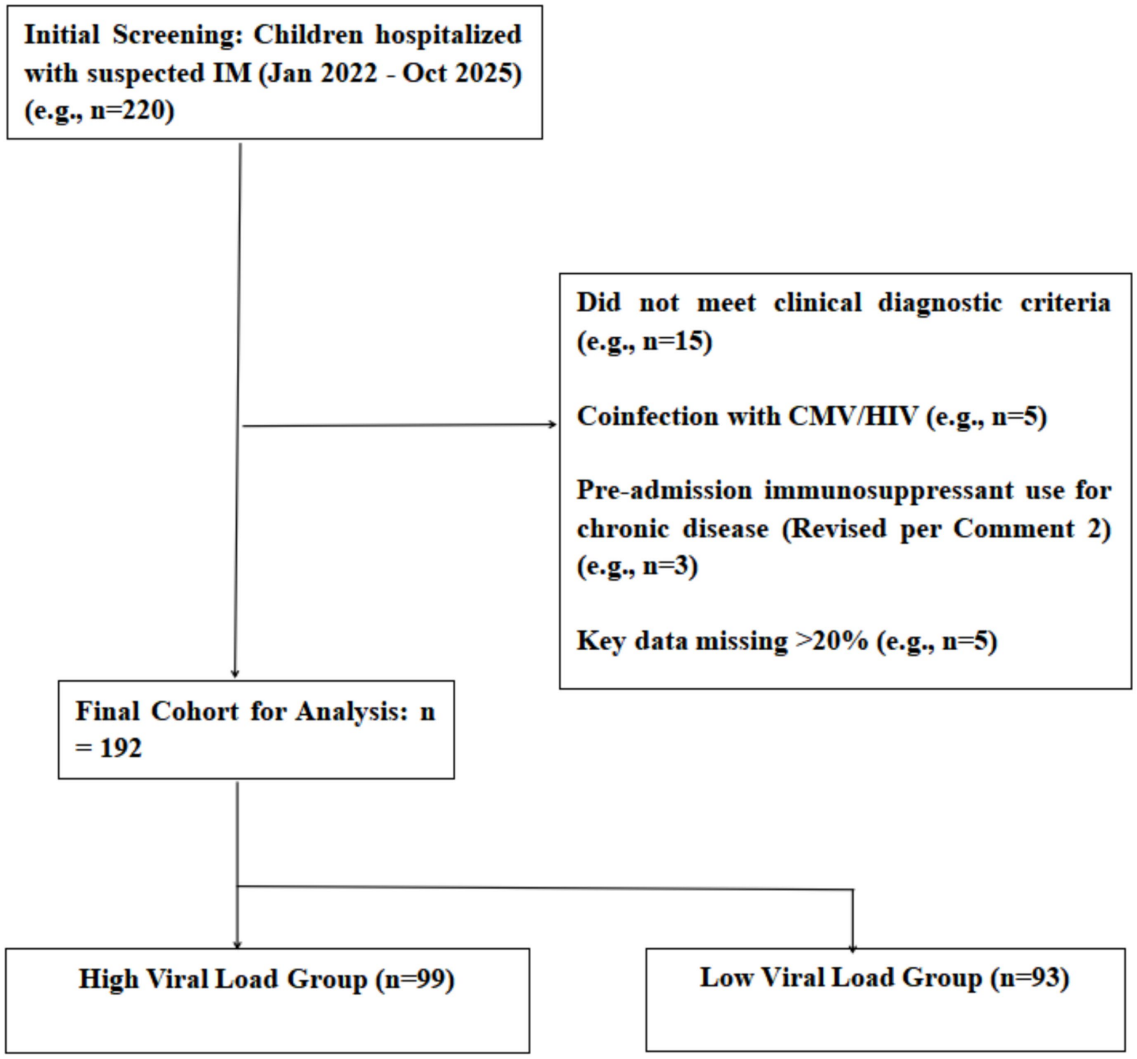
Flowchart of patient selection detailing the application of inclusion and exclusion criteria leading to the final study cohort of 192 children.

This retrospective study consecutively screened all pediatric patients hospitalized in our department with a diagnosis suspected to be IM during a predefined study period (January 2022 to October 2025). The final cohort represents a subset of children with primary symptomatic EBV infection who met stringent clinical and laboratory criteria for IM, as detailed below. The recruitment rate of approximately 4 cases per month from our tertiary center is consistent with the expected frequency of clinically significant IM requiring hospitalization after applying rigorous diagnostic filters. All patients underwent systematic clinical evaluation and laboratory investigations. The participant selection process is summarized in Figure 1 (descriptive text: initial identification of suspected IM cases through electronic medical record system diagnostic codes was followed by independent chart review by two senior pediatricians to confirm cases meeting clinical and serological diagnostic criteria; final inclusion in the cohort was determined based on completeness of viral load data and exclusion criteria).

This retrospective study consecutively screened all pediatric patients hospitalized in our department with a diagnosis suspected to be IM during a predefined study period (January 2022 to October 2025). After applying the inclusion and exclusion criteria (see Figure 1 for the participant flowchart), 192 children were included in the final analysis. Stratification was based on the median initial EBV DNA load (3.0 log_10_ copies/mL) measured at admission. Patients with values above this median were assigned to the high viral load group (*n* = 99; 51 males, 48 females; age range: 1–12 years), and those below to the low viral load group (*n* = 93; 52 males, 41 females; with a comparable age distribution). Stratification was based on the median initial EBV DNA load (3.0 log_10_ copies/mL) measured at admission. Patients with values above this median were assigned to the high viral load group (*n* = 99; 51 males, 48 females; age range: 1–12 years), and those below to the low viral load group (*n* = 93; 52 males, 41 females; with a comparable age distribution).

### 2.2 Inclusion and exclusion criteria

Stringent inclusion and exclusion criteria were applied to ensure cohort homogeneity and data reliability.

#### 2.2.1 Inclusion criteria

1. Age between 1 and 14 years.
2. Meeting the classic clinical diagnostic criteria for IM (1), namely the concurrent presence of the triad: fever (axillary temperature ≥38.0 °C, lasting ≥3 days), pharyngotonsillitis (with or without exudative tonsillitis), and cervical lymphadenopathy (lymph node diameter ≥1 cm).
3. Patients should meet the following criteria: at least two of the three clinical manifestations—fever, pharyngitis, and lymphadenopathy—concurrent with either a proportion of atypical lymphocytes ≥10% in laboratory testing or a positive serological result for anti-Epstein–Barr virus capsid antigen (EBV-VCA) immunoglobulin M (IgM) antibodies.

#### 2.2.2 Exclusion criteria

1. Coinfection with other active viruses, evidenced by seropositivity for cytomegalovirus (CMV) or human immunodeficiency virus (HIV).
2. Administration of systemic glucocorticoids or immunosuppressants for chronic conditions (e.g., autoimmune diseases, asthma) prior to admission.
3. Presence of congenital or acquired immunodeficiency disorders, malignancies, or hematological diseases.
4. Admission to or primary management in the intensive care unit (ICU) at the onset of hospitalization. This criterion aims to ensure cohort homogeneity regarding the standard ward-based management pathway against which the primary outcome (length of stay) is measured.
5. Missing more than 20% of key clinical data (e.g., temperature records, critical laboratory indices) in the electronic medical records.

These stringent criteria were designed to identify children with symptomatic primary EBV infection (IM) of sufficient clinical severity for hospitalization, rather than all children with EBV exposure or mild infection. Consequently, the final cohort size reflects the proportion of pediatric EBV infections that progress to this more defined and clinically significant phenotype within our institutional setting.

### 2.3 Equipment and materials

All laboratory assays were performed in the hospital’s central laboratory, with routine equipment maintenance and calibration adhering to standard operating procedures.

#### 2.3.1 Viral load quantification

EBV DNA load was quantified using a laboratory-developed real-time PCR assay on the QuantStudio 5 System (Applied Biosystems, United States). Nucleic acid was extracted from 200 μL of plasma using the QIAamp DSP Virus Kit (Qiagen, Germany) according to the manufacturer’s instructions. Primers and a TaqMan probe were designed to target a conserved region of the EBV latent membrane protein 1 (LMP1) gene. The primer and probe sequences were as follows:

Forward primer (LMP1-F): 5′-CCAGACAGCAGCCAACAATTGTC-3′.

Reverse primer (LMP1-R): 5′-GGTAGAAGACCCCCTCTTAC-3′.

Probe (LMP1-Probe): 5′-FAM-GAACAGCACAATTCCAAGGAACAATGCCTG-BHQ1-3′.

The amplicon size was 129 bp. The 20 μL PCR reaction contained 10 μL of 2 × QuantiNova Probe PCR Master Mix (Qiagen), 0.8 μL of each primer (10 μM), 0.4 μL of probe (10 μM), and 5 μL of extracted DNA. Thermal cycling conditions were: initial denaturation at 95 °C for 15 min, followed by 45 cycles of 95 °C for 30 s (denaturation), 56 °C for 30 s (annealing), and 72 °C for 30 s (extension). A standard curve was generated using serial 10-fold dilutions of a plasmid containing the LMP1 target fragment (range: 1.0 × 10^2^ to 1.0 × 10^7^ copies/mL). Each run included negative (no template) and positive controls. The lower limit of detection was 400 copies/mL. Results were converted to log_10_ copies/mL for analysis.

#### 2.3.2 Serological testing

Anti-EBV antibodies (anti-VCA IgM, anti-VCA IgG, anti-EBNA IgG) were detected using enzyme-linked immunosorbent assay (ELISA) kits (Euroimmun Medizinische Labordiagnostika AG, Germany), strictly following the manufacturer’s protocols. Absorbance was read using a Multiskan FC microplate reader (Thermo Fisher Scientific, United States). Results were interpreted as positive, negative, or equivocal based on the kit-specific cut-off values.

#### 2.3.3 Biochemical and hematological analyses

Liver function parameters, including alanine aminotransferase (ALT) and aspartate aminotransferase (AST), were measured using a 7,600 automatic biochemical analyzer (Hitachi, Japan). Heterophile antibodies were screened using the standard Paul-Bunnell test (sheep red blood cell agglutination). Complete blood cell counts and differentials were performed using the XN-9000 automated hematology analyzer (Sysmex, Japan).

### 2.4 Study procedures

This retrospective cohort study was conducted as follows:

#### 2.4.1 Exposure definition and grouping

The exposure variable was the first EBV DNA load measured within 24 h of admission. To facilitate clinical interpretation and ensure balanced group sizes, the overall median initial viral load of the cohort (3.0 log_10_ copies/mL) was used as the cutoff, rather than absolute thresholds like 10^3^ or 10^4^ copies/mL. Patients were categorized into a high viral load group (>3.0 log_10_ copies/mL) and a low viral load group (<3.0 log_10_ copies/mL) for comparative analysis.

#### 2.4.2 Data collection and extraction

A data extraction team, comprising one attending physician and one research nurse, was established. Using a pre-designed standardized case report form (CRF), data were extracted in a blinded manner (i.e., unaware of group assignment) from the Hospital Information System (HIS) and Laboratory Information System (LIS). Extracted data included: (1) Demographics: age, sex; (2) Clinical data: symptoms and signs at admission, occurrence of complications, therapeutic agents (specifying type, dosage, start and end dates for glucocorticoids and antimicrobials), total length of hospital stay; (3) Laboratory data: serial EBV DNA loads (at admission, during treatment, and pre-discharge), EBV serological antibody titers, liver function tests, complete blood counts, and heterophile antibody results.

#### 2.4.3 Quality control

To ensure data extraction accuracy and assay stability, the following measures were implemented: Firstly, another physician not involved in the initial data entry randomly reviewed 10% of the cases (*n* = 19) to verify the consistency of key variables. Secondly, 5% of serum samples (approx. 10) were randomly selected for re-testing of EBV DNA load to assess assay reproducibility. Agreement was evaluated using Kappa statistics, which indicated excellent consistency (Kappa = 0.92).

### 2.5 Outcome measures

Based on the study objectives, the following seven primary outcome measures were established, representing classic clinical and laboratory parameters for assessing IM severity and prognosis:

#### 2.5.1 Primary outcome

Total length of hospitalization, defined as the actual calendar days from admission to discharge. This is a key indicator of overall disease burden and healthcare resource utilization.

#### 2.5.2 Virological outcome

Dynamic changes in EBV DNA load. The EBV DNA load (log_10_ copies/mL) at admission (baseline) and pre-discharge (typically 1-2 days before discharge), as well as the magnitude of its decrease, were recorded and compared between groups. This directly reflects the activity of viral replication and its response to treatment.

#### 2.5.3 Serological outcomes

Included anti-VCA IgM/IgG titers and anti-EBNA IgG seropositivity rate. Changes in titers from admission to pre-discharge were recorded. Anti-VCA IgM is a marker of acute infection, and its dynamics reflect the course of acute illness; the appearance and pattern of anti-VCA IgG and anti-EBNA IgG help confirm primary infection and assess the establishment of immune response. Titer results were recorded as S/CO values (sample absorbance/cut-off absorbance) from ELISA or quantitative titers if applicable.

#### 2.5.4 Biochemical and immunological outcomes

Included liver function tests and heterophile antibody. Peak values of ALT and AST (U/L) during hospitalization were recorded as direct indicators of IM-associated hepatitis severity. The positivity rate (%) for heterophile antibody at admission was recorded as a classic, albeit non-specific, supportive diagnostic marker for IM.

#### 2.5.5 Complication outcomes

Complications occurring during hospitalization and confirmed by objective examinations were recorded. These primarily included: (1) Splenomegaly: confirmed by abdominal ultrasonography, defined as spleen thickness exceeding the upper limit of normal for age and sex or a longitudinal diameter >13 cm; (2) Respiratory obstruction: required clear clinical documentation (e.g., stridor, dyspnea) and confirmation by an otolaryngologist assessment; (3) Anemia: defined as a hemoglobin (Hb) concentration below 100 g/L.

#### 2.5.6 Treatment-related outcomes

Included the rate of systemic glucocorticoid use (%) and the total duration (days) of intravenous/oral antimicrobial administration. The indication for glucocorticoid use (e.g., severe pharyngeal edema, persistent high fever) and specific agents (e.g., prednisone, methylprednisolone) were recorded. The duration of antimicrobial use only included agents administered for treating or preventing secondary bacterial infections.

#### 2.5.7 Hematological outcome

The proportion (%) of atypical lymphocytes in peripheral blood smears. Two experienced technicians from the hospital’s laboratory department performed blinded microscopic examinations on blood smears obtained during the peak illness phase (typically 3–7 days post-admission). They counted at least 100 white blood cells, calculating the percentage of atypical lymphocytes (characterized by enlarged cell size, basophilic cytoplasm, and dispersed nuclear chromatin—activated lymphocytes). The average of their counts was used as the observed value for each patient. This is a characteristic hematological finding in IM.

### 2.6 Admission and discharge criteria

To provide context for the primary outcome (length of hospitalization), the institutional practice guidelines for admission and discharge during the study period are outlined.

#### 2.6.1 Admission criteria

Hospitalization was typically indicated for children with IM presenting with one or more of the following: (1) Persistent high fever (≥39.0 °C) refractory to outpatient antipyretics for >48 h; (2) Inability to maintain adequate oral hydration; (3) Significant pharyngotonsillar involvement with concern for potential airway compromise; (4) Biochemical evidence of moderate to severe hepatitis (e.g., ALT/AST > 5 times the upper limit of normal); (5) Clinical or ultrasound evidence of splenomegaly with activity restrictions advised; or 6) Development of suspected complications (e.g., hematologic abnormalities).

#### 2.6.2 Discharge criteria

Patients were considered medically fit for discharge upon meeting all of the following: (1) Afebrile for at least 24 h without antipyretics; (2) Able to tolerate adequate oral intake; (3) Showing a clear downward trend in liver transaminases (ALT/AST); (4) Absence of signs of acute complications; and (5) Parents/caregivers received appropriate education regarding activity restriction (especially contact sports to prevent splenic injury) and signs of potential late complications.

### 2.7 Statistical analysis

All statistical analyses were performed using IBM SPSS Statistics software version 26.0. A two-sided *p*-value <0.05 was considered statistically significant.

First, the normality of continuous variables was assessed using the Shapiro–Wilk test. Normally distributed data are presented as mean ± standard deviation (*x̄* ± *s*) and compared between groups using the independent samples *t*-test. Non-normally distributed data are presented as median and interquartile range (Median, IQR) and compared using the Mann–Whitney *U* test.

Second, categorical data are presented as frequencies and percentages (*n*, %). Group comparisons were made using the Chi-square (*χ*^2^) test or Fisher’s exact test when expected frequencies were less than 5.

For the primary outcome, length of hospitalization, survival analysis methods were employed. “Discharge” was defined as the event. Kaplan–Meier survival curves were plotted, and the Log-rank test was used to compare the distribution of time to discharge between the two groups. To further control for potential confounding factors, variables with *p* < 0.1 in univariate analysis and those deemed clinically important (e.g., age, sex, peak ALT, occurrence of splenomegaly, and glucocorticoid use) were incorporated into a multivariate Cox proportional hazards regression model. Hazard ratios (HR) and their 95% confidence intervals (CI) were calculated to identify independent factors influencing the length of hospitalization.

For the small amount of missing data present in this study (missingness for key variables was below 5%), multiple imputation was used to generate five complete datasets. Results from analyses on these datasets were pooled. Post-hoc power analysis was conducted for the primary outcome (length of hospital stay) using the observed effect size between groups. With a two-sided *α* of 0.05, the study had >80% power to detect the observed difference. All analyses report specific test statistics (e.g., *t*, *U*, *χ*^2^, and HR values) and exact *p*-values.

## 3 Results

### 3.1 Comparison of baseline characteristics

**Figure 2:**
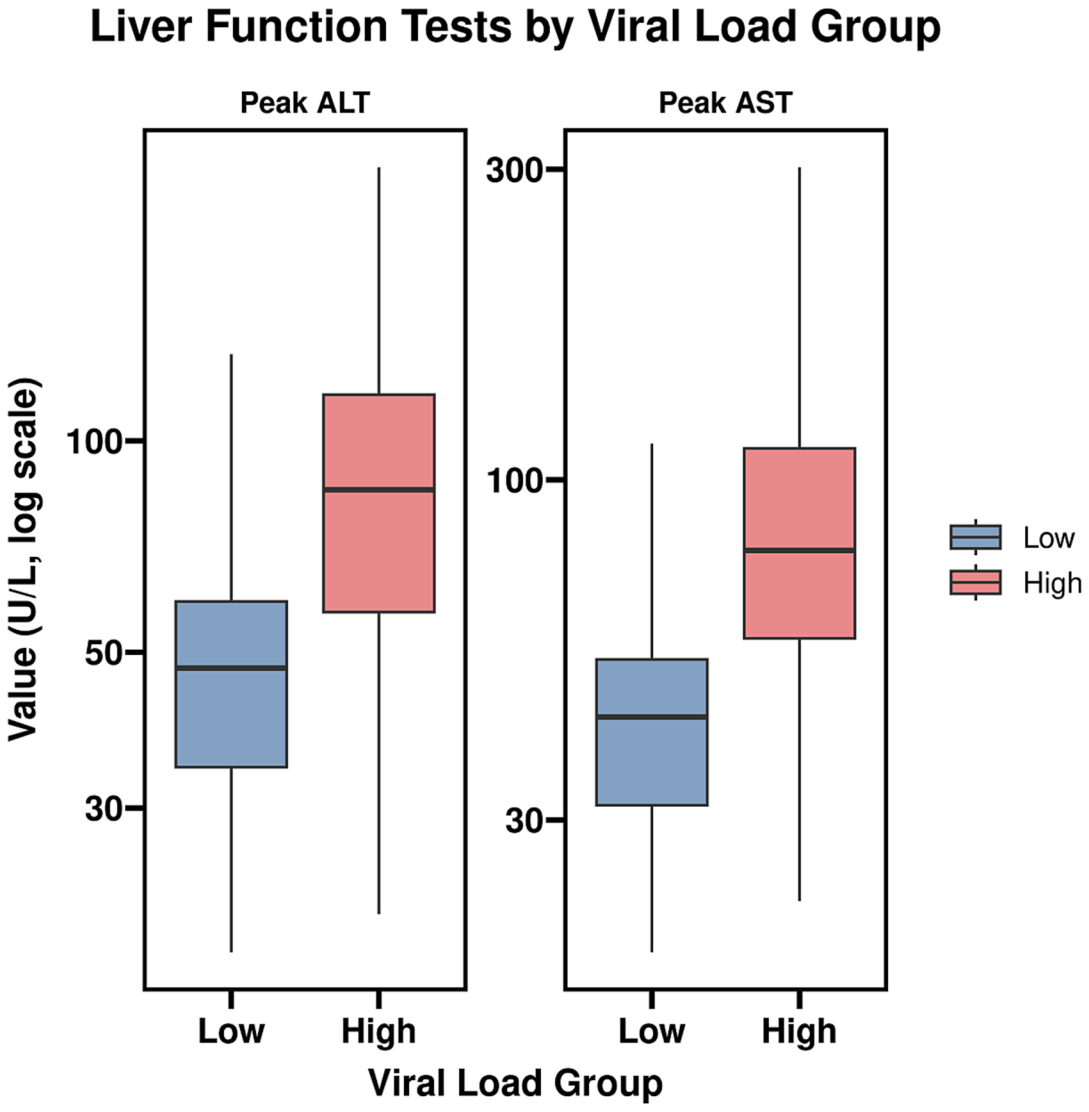
Liver function tests by viral load group. Box plots showing the distribution of peak alanine aminotransferase (ALT) and aspartate aminotransferase (AST) levels in the high (*n* = 99) and low (*n* = 93) viral load groups. The high viral load group demonstrated significantly elevated median peak alanine aminotransferase (ALT) (*p* < 0.001) peak aspartate aminotransferase (AST) (*p* < 0.001).

Analysis of baseline demographic and clinical characteristics between the high and low viral load groups revealed significant differences in specific laboratory markers. The high viral load group demonstrated significantly elevated median peak alanine aminotransferase (ALT) (*p* < 0.001), peak aspartate aminotransferase (AST) (*p* < 0.001), peak gamma-glutamyl transferase (GGT) (*p* < 0.001), and proportion of atypical lymphocytes (*p* = 0.036). No statistically significant differences were found between the two groups regarding age, sex distribution, admission body temperature, duration of symptoms prior to admission, or anti-EBNA seropositivity at diagnosis (all *p* > 0.05). Detailed comparative data are presented in Table 1 and Figure 2.

**Table 1:** Comparison of baseline characteristics between high and low viral load groups.

Characteristic	High viral load group (*n* = 99)	Low viral load group (*n* = 93)	*p*-value
Age (years), median (IQR)	5.42 (3.78, 7.15)	5.18 (3.60, 6.94)	0.517
Sex, *n* (%)			0.589
Male	51 (51.52)	52 (55.91)	
Female	48 (48.48)	41 (44.09)	
Admission temperature (°C), median (IQR)	38.9 (38.4, 39.4)	38.7 (38.2, 39.3)	0.387
Duration of symptoms (days), median (IQR)	4.0 (3.0, 5.0)	4.0 (3.0, 5.0)	0.835
Peak ALT (U/L), median (IQR)	86.34 (52.17, 135.42)	45.26 (32.15, 68.91)	<0.001
Peak AST (U/L), median (IQR)	78.91 (47.33, 120.65)	40.58 (29.74, 55.12)	<0.001
Atypical lymphocyte proportion (%), median (IQR)	18.35 (12.40, 25.60)	15.20 (10.10, 21.85)	0.036
Anti-VCA IgM positive at diagnosis, *n* (%)	87 (87.88)	84 (80.32)	0.588
Anti-EBNA positive at diagnosis, *n* (%)	12 (12.12)	10 (10.75)	0.764
Peak GGT (U/L), median (IQR)	52.10 (28.40, 89.70)	31.50 (19.20, 54.80)	<0.001

IQR, interquartile range; ALT, alanine aminotransferase; AST, aspartate aminotransferase; anti-VCA, anti-viral capsid antigen; anti-EBNA, anti-Epstein–Barr nuclear antigen.

### 3.2 Treatment and clinical outcomes

Comparative analysis of therapeutic interventions and clinical outcomes indicated notable disparities between the groups. The utilization rate of systemic glucocorticoids was significantly higher in the high viral load group (*χ*^2^ = 9.663, *p* = 0.002). Correspondingly, the total length of hospital stay was markedly prolonged in the high viral load group compared to the low viral load group (*U* = 2865.000, *p* < 0.001). In contrast, the rate of antimicrobial administration and the median duration of antimicrobial therapy did not differ significantly between the two groups (*p* > 0.05). These findings are summarized in Table 2 and Figure 2.

**Table 2:** Comparison of treatment and clinical outcomes between groups.

Outcome measure	High viral load group (*n* = 99)	Low viral load group (*n* = 93)	*p*-value
Glucocorticoid use, *n* (%)	50 (50.51)	27 (29.03)	0.002
Antimicrobial use rate, *n* (%)	75 (75.76)	72 (77.42)	0.786
Duration of antimicrobial use (days), median (IQR)	6.0 (5.0, 7.0)	5.0 (4.0, 7.0)	0.302
Hospital stay (days), median (IQR)	8.50 (7.00, 10.00)	6.00 (5.00, 8.00)	<0.001

### 3.3 Virological and serological dynamics

Table 3 compares the seropositivity rate of anti-VCA IgM at admission between the two groups. The proportion of patients positive for anti-VCA IgM was similar in the high viral load group (65.66%) and the low viral load group (64.52%), with no statistically significant difference (*p* = 0.869). These findings indicate a distinct dissociation between the magnitude of EBV viremia and the prevalence of IgM seropositivity at initial presentation.

**Table 3:** Comparison of virological and serological parameters at admission between high and low viral load groups.

Parameter	High viral load group (*n* = 99)	Low viral load group (*n* = 93)	*p*-value
Anti-VCA IgM positive, *n* (%)	65 (65.66)	60 (64.52)	0.869

IQR, interquartile range; GMT, geometric mean titer.

### 3.4 Incidence of complications

The analysis of complication rates demonstrated a higher disease burden associated with elevated viral load. The overall incidence of developing any complication during hospitalization was significantly greater in the high viral load group (*χ*^2^ = 13.505, *p* < 0.001). Specifically, the occurrence of splenomegaly, confirmed by ultrasonography, was markedly higher in the high viral load group (*χ*^2^ = 11.57, *p* = 0.001). The incidence rates of respiratory obstruction and anemia (Hb < 100 g/L) did not show statistically significant differences between the two groups (*p* > 0.05). These results are detailed in Table 4.

**Table 4:** Comparison of complication incidence between groups [*n* (%)].

Complication	High viral load group (*n* = 99)	Low viral load group (*n* = 93)	*χ*^2^ value	*p*-value
Any complication	59 (59.60)	31 (33.33)	13.505	<0.001
Splenomegaly	43 (43.43)	19 (20.43)	11.57	0.001
Respiratory obstruction	11 (11.11)	5 (5.38)	2.082^*^	0.17
Anemia (Hb < 100 g/L)	15 (15.15)	9 (9.68)	1.363	0.243

^*^Fisher’s exact test.

### 3.5 Multivariate Cox regression analysis of factors influencing hospital stay

**Figure 3:**
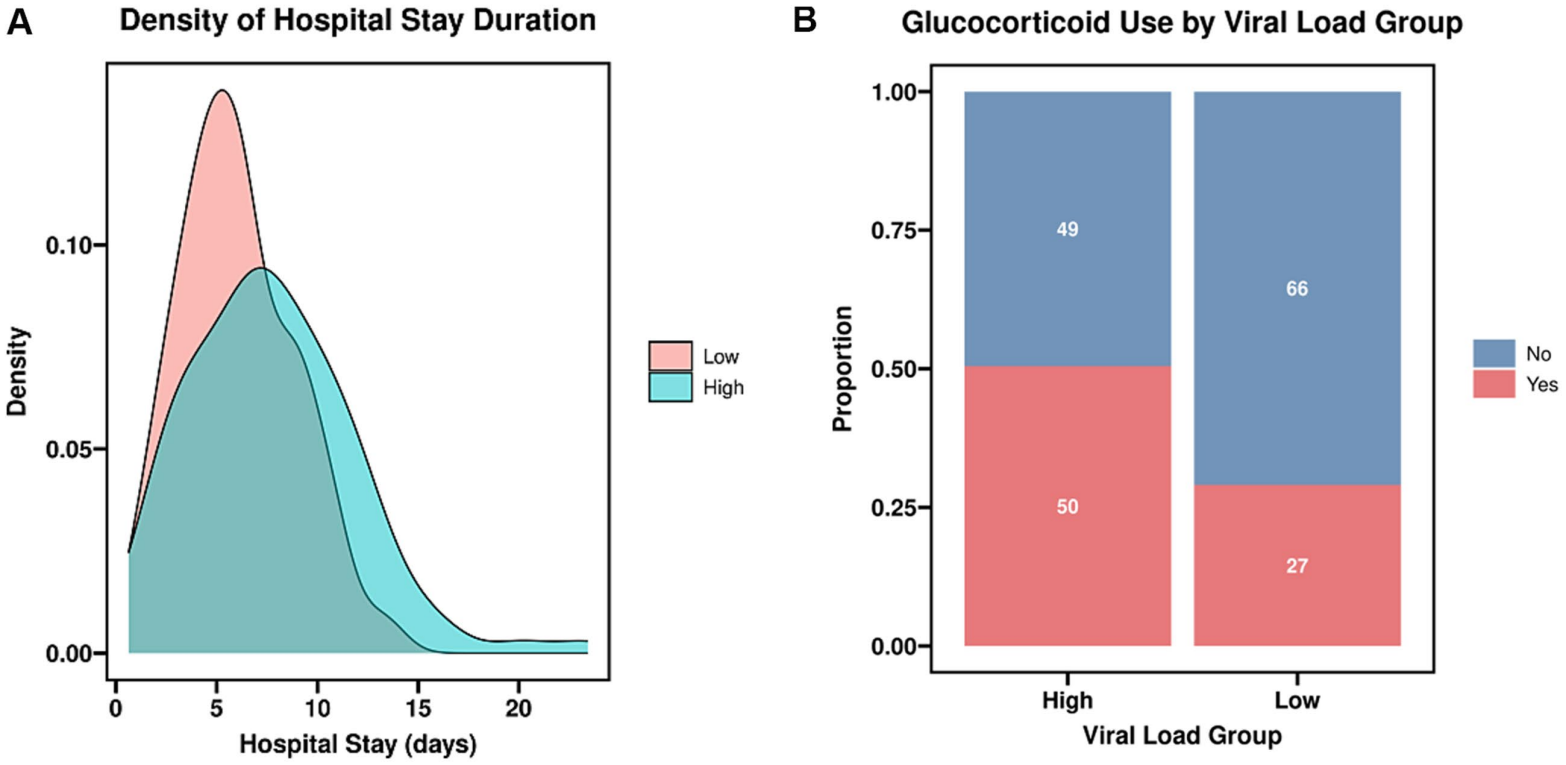
**(A)** Density of hospital stay duration. The total length of hospital stay was markedly prolonged in the high viral load group compared to the low viral load group (*p* < 0.001). **(B)** Glucocorticoid use by viral load group. The utilization rate of systemic glucocorticoids was significantly higher in the high viral load group (*p* = 0.002).

**Figure 4:**
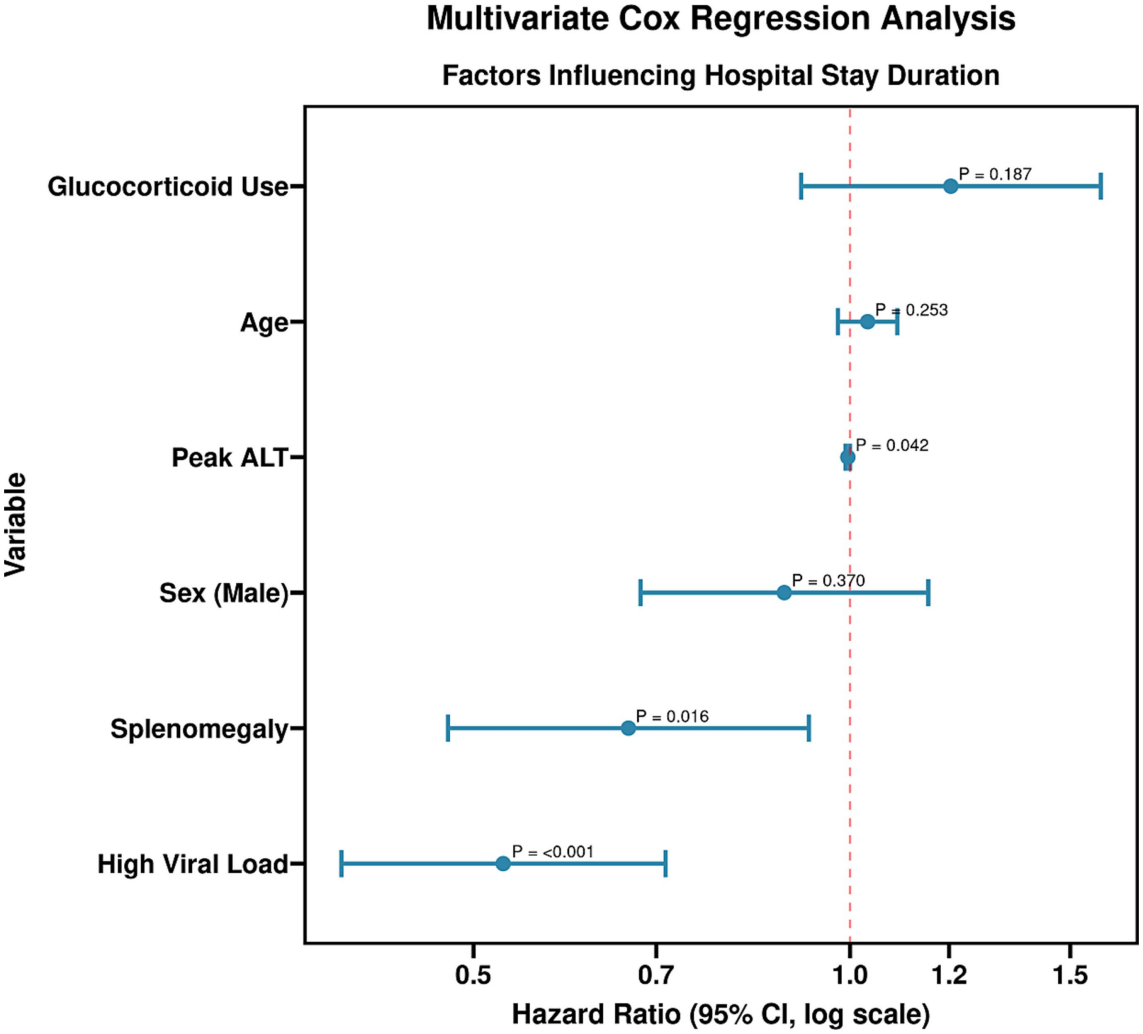
Multivariate Cox regression analysis. The analysis identified high viral load (HR = 0.528, 95% CI: 0.392–0.712; *p* < 0.001) and splenomegaly (HR = 0.665, 95% CI: 0.477–0.927; *p* = 0.016) as significant independent factors associated with a longer time to discharge, indicated by hazard ratios less than 1. Peak ALT level also showed a statistically significant but minimal effect (HR = 0.996, *p* = 0.042). Age, sex, and glucocorticoid use were not independent significant predictors in this model.

To identify independent predictors of the length of hospitalization, a multivariate Cox proportional hazards regression model was constructed. The dependent variable was time to discharge. Covariates entered into the model included high viral load status (yes vs. no), presence of splenomegaly (yes vs. no), age (years), sex (male vs. female), peak ALT level (U/L), and glucocorticoid use (yes vs. no). The analysis identified high viral load (HR = 0.528, 95% CI: 0.392–0.712; *p* < 0.001) and splenomegaly (HR = 0.665, 95% CI: 0.477–0.927; *p* = 0.016) as significant independent factors associated with a longer time to discharge, indicated by hazard ratios less than 1. Peak ALT level also showed a statistically significant but minimal effect (HR = 0.996, *p* = 0.042). Age, sex, and glucocorticoid use were not independent significant predictors in this model. The overall model was statistically significant (Likelihood ratio *χ*^2^ = 34.572, *p* < 0.001). The results of the Cox regression analysis are presented in Table 5 and Figures 3, 4.

**Table 5:** Multivariate Cox proportional hazards regression analysis of factors influencing hospital stay.

Variable	*b* value	SE	Wald *χ*^2^	*p*-value	HR (95% CI)
High viral load (yes vs. no)	−0.639	0.151	17.869	<0.001	0.528 (0.392–0.712)
Splenomegaly (yes vs. no)	−0.408	0.17	5.757	0.016	0.665 (0.477–0.927)
Age (years)	0.032	0.028	1.307	0.253	1.033 (0.978–1.091)
Sex (male vs. female)	−0.121	0.135	0.804	0.37	0.886 (0.680–1.155)
Peak ALT (U/L)	−0.004	0.002	4.139	0.042	0.996 (0.992–1.000)
Glucocorticoid use (yes vs. no)	0.186	0.141	1.74	0.187	1.204 (0.914–1.587)

**Figure 5:**
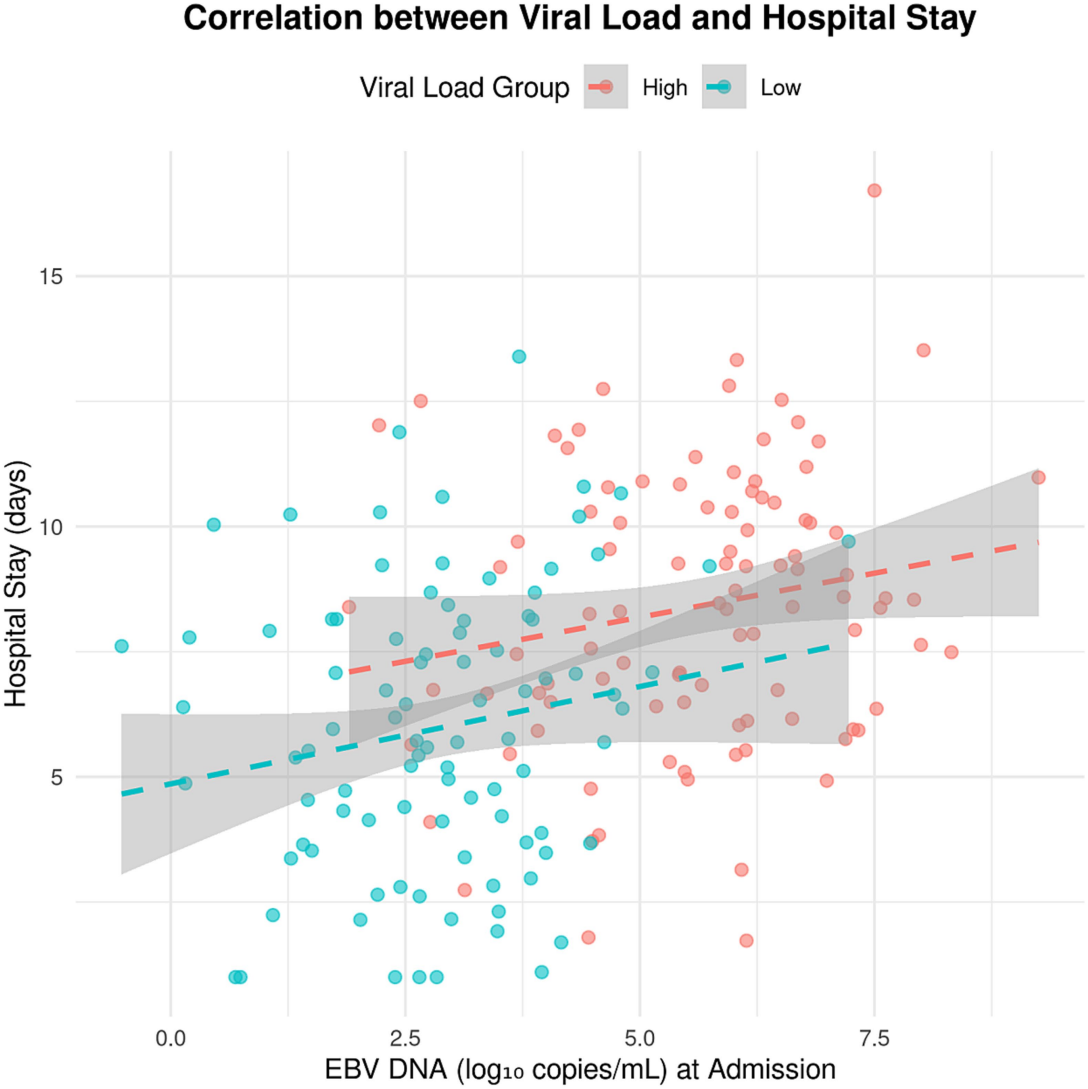
Correlation between viral load and hospital stay. This scatter plot visualizes the relationship between Epstein–Barr virus (EBV) DNA levels at hospital admission (*x*-axis, log_10_ copies/mL) and duration of hospital stay (*y*-axis, days) in patients with EBV-associated infectious mononucleosis. Patients were classified into two groups: High viral load (pink circles, defined as ≥4 log_10_ copies/mL) and low viral load (teal circles, <4 log_10_ copies/mL). Dashed lines represent linear regression trend lines for each group, and the gray shaded area denotes the 95% confidence interval of the regression. EBV DNA was quantified using real-time PCR (assay details are provided in the Methods section).

## 4 Discussion

Epstein–Barr virus infection represents a common childhood illness characterized by considerable heterogeneity in its clinical presentation. The relationship between viral load and disease severity has emerged as a focal point of contemporary research (16). By systematically comparing clinical features, treatment responses, and prognostic indicators between children stratified by viral load, our study found that the high viral load group exhibited significantly greater hepatic injury, higher risk of complications, and increased utilization of healthcare resources compared to the low viral load group (17). These findings not only affirm the clinical predictive value of viral load but also provide crucial data for establishing a viral load-based risk stratification framework, holding significant implications for guiding individualized clinical management.

In the analysis of baseline characteristics, the high viral load group demonstrated markedly elevated levels of ALT, AST, and GGT, along with a higher proportion of atypical lymphocytes. The significant elevation in GGT, a marker that can reflect both hepatocellular damage and cholestasis, further corroborates the multifaceted nature of EBV-associated liver injury and strengthens the association between high viral replication and the severity of hepatic involvement. The concurrent elevation of these three parameters strongly suggests a close pathophysiological link between active viral replication and the dual processes of hepatic injury and immune system activation (18). Previous studies have indicated that EBV can directly infect hepatocytes, causing cytopathic effects, while simultaneously triggering a robust T-cell immune response. This aligns with our observations of elevated liver enzymes and increased atypical lymphocytes (19). The underlying mechanism likely involves a dual pathway of direct viral cytotoxicity and immune-mediated hepatic tissue damage, a process that may be more pronounced with higher viral loads. The median age of our cohort (5 years) is younger than the classic peak of IM in adolescents commonly described in Western populations. This likely reflects the epidemiological pattern of earlier primary EBV infection in children within our region, where a higher proportion of primary infections in early childhood manifest with sufficient symptomatology to warrant hospitalization. Our stringent diagnostic criteria—requiring the clinical triad plus either significant atypical lymphocytosis or positive EBV-VCA IgM—were specifically designed to identify true acute symptomatic IM rather than incidental EBV detection or reactivation in asymptomatic carriers. This strengthens the validity of our cases as representative of acute, clinically significant primary infection in this younger age group.

Regarding treatment, the significantly higher rate of glucocorticoid use and the markedly prolonged hospital stay in the high viral load group indicate that these patients required more intensive immunomodulatory intervention and experienced a more protracted clinical course (20). This phenomenon may be attributable to the more intense cytokine storm and more extensive tissue damage induced by a high viral burden, necessitating stronger immunomodulatory therapy to control disease progression (21). In contrast to prior studies, our research not only confirms the association between viral load and treatment intensity but also, through multidimensional data analysis, clarifies the significant impact of viral load on healthcare resource consumption, providing empirical evidence for clinical resource allocation.

Virological dynamic monitoring revealed that although EBV DNA levels decreased significantly post-treatment in both groups, the high viral load group maintained higher levels both at admission and discharge. This suggests that the baseline viral load may influence the rate of viral clearance (22). The inter-group difference in anti-EBNA seropositivity rates further indicates that a high viral load might prolong the state of active viral replication by affecting the immunoregulatory processes involved in the transition to viral latency (23). The deeper mechanism may involve the modulation of host immune response efficacy by viral load, where a high-load state could delay the establishment of effective immune control through certain immunosuppressive mechanisms.

Analysis of complications demonstrated a significantly higher overall incidence in the high viral load group, with splenomegaly being the most prominent. This reflects a positive correlation between viral load and disease severity (24). The increased incidence of splenomegaly may stem from the clonal proliferation of B lymphocytes due to direct viral infection, coupled with subsequent immune activation and inflammatory cell infiltration (25). Compared to previous research, our study quantifies the risk relationship between viral load and specific complications using a large sample, offering a more precise predictive marker for the early identification and intervention of high-risk children.

Multivariate Cox regression analysis further confirmed that a high viral load is an independent risk factor for prolonged hospitalization, highlighting its central role in prognostic assessment (26). Splenomegaly and elevated ALT were also significantly associated with a longer clinical course, likely because they reflect two critical pathophysiological processes: the degree of immune system activation and the severity of hepatic injury, respectively (27). In contrast to prior studies focusing primarily on clinical indicators, our research, by constructing a multivariate predictive model, quantifies for the first time the independent contribution of viral load to influencing hospitalization duration, providing a novel perspective for clinical prognosis evaluation.

This study has several limitations. The single-center design and retrospective data collection may introduce selection bias, and not all potential confounding factors could be accounted for. Furthermore, due to the retrospective nature of the study and the fact that thyroid function testing is not part of the routine clinical workup for uncomplicated infectious mononucleosis in our center, data on thyroid parameters (e.g., TSH, FT3, FT4) were not systematically available and could therefore not be analyzed. Future prospective studies should consider incorporating thyroid function tests to explore potential subclinical endocrine involvement or the impact of the systemic inflammatory response on the hypothalamic–pituitary-thyroid axis during acute EBV infection. Future prospective, multicenter studies are warranted to validate the predictive utility of viral load and to further explore its association with specific immunological markers. Furthermore, the degree of standardization in treatment protocols may affect the generalizability of our findings, necessitating validation in different healthcare settings. Third, we used the total length of hospitalization as the primary outcome measure. While this is an objective indicator of healthcare resource utilization and reflects the overall time required for clinical stabilization, we acknowledge its inherent limitations. LOS can be influenced by non-clinical factors such as physician discretion, institutional protocols, weekend discharge patterns, and parental anxiety. Importantly, the possibility that knowledge of a high EBV DNA load may have subconsciously influenced clinicians to prolong observation or initiate immunomodulatory therapy (e.g., corticosteroids) cannot be entirely ruled out, potentially creating a self-fulfilling prophecy bias. Our multivariate Cox model attempted to adjust for some markers of disease severity (e.g., peak ALT, splenomegaly, steroid use), but residual confounding likely persists. Therefore, while our findings demonstrate a robust association between high viral load and prolonged LOS, this outcome should be interpreted as a composite measure of disease severity and its management within a specific healthcare system, rather than a pure biological marker of disease severity itself. Future prospective studies incorporating more direct and specific measures of disease resolution (e.g., standardized clinical severity scores at discharge) would be valuable. Fourth, the sample size of 192 patients, while representing the complete cohort of eligible cases meeting our stringent criteria within the defined retrospective period, should be interpreted with nuance. A post-hoc power analysis confirmed that the study was adequately powered (>80%) to detect the statistically significant and clinically meaningful differences observed in the primary outcome (length of hospital stay) between the high and low viral load groups. However, we acknowledge that this sample size may limit the statistical power and precision of effect size estimates for some secondary outcome measures and precludes more granular subgroup analyses (e.g., stratifying by age or specific complication types). Therefore, while our primary conclusions are robust, the findings for secondary endpoints should be considered exploratory and warrant validation in larger, ideally multicenter, prospective cohorts. Fifth, an important limitation pertains to patient selection. To maintain a cohort with comparable management pathways for the analysis of hospitalization duration, we excluded patients who were admitted to or primarily managed in the ICU. As the reviewer astutely noted, this may have introduced a selection bias by excluding the most severe cases, for whom the association between viral load and disease severity might be most pronounced. Consequently, our findings on the viral load-severity relationship are derived from and applicable to the spectrum of disease severity requiring general pediatric inpatient care but not critical care. This limits the generalizability of our results to the most critically ill children with EBV-IM. Future studies specifically designed to include patients across all care settings, including the ICU, are necessary to fully characterize the predictive value of viral load across the entire severity continuum.

In summary, this study demonstrates that a high EBV DNA load at admission is an independent predictor of more severe hepatic injury, increased complication rates (particularly splenomegaly), greater requirement for immunomodulatory therapy, and prolonged hospitalization in children with IM. These findings robustly support the integration of routine viral load quantification into the initial clinical assessment to enable early risk stratification. Looking toward future prospects, our results provide a strong rationale for prospective, multicenter studies to validate and refine viral load thresholds for guiding clinical decisions, such as the initiation of corticosteroids or the need for prolonged monitoring. Furthermore, this work lays a critical foundation for subsequent mechanistic research aimed at elucidating the dynamic interplay between viral replication kinetics and host immune responses. Investigating whether early viral load trends or specific host immune signatures (e.g., cytokine profiles) can enhance the predictive value of a single baseline measurement represents a key next step toward developing personalized management strategies for pediatric EBV infection.

## Data Availability

The dataset generated and analyzed during this study is not publicly available due to patient privacy and confidentiality protections. The data contains sensitive clinical and laboratory information from pediatric patients treated at Anhui Children’s Hospital. All data were anonymized in accordance with institutional requirements and the Declaration of Helsinki, but the dataset includes protected health information that cannot be shared publicly without violating patient privacy regulations and Ethics Committee stipulations (Approval No. EYLL-2025-106). Requests to access the datasets should be directed to Yuan Xu; xyuan0324@163.com.
