# Correction: Association between Epstein–Barr viral load and duration of hospitalization in pediatric infectious mononucleosis: a single-center retrospective study

**DOI:** 10.3389/fmed.2026.1879795

**Published:** 2026-06-09

**Authors:** Zhi Yang, Zongtao Ma, Qingfeng Fang, Biquan Chen, Yuan Xu

**Affiliations:** 1 Department of Pediatric Infectious Disease, Anhui Provincial Children's Hospital, Hefei, China; 2 Department of Pediatrics, Lixin Hospital of Chinese Medicine, Bozhou, China; 3 Department of Pediatric Nephrology, Anhui Provincial Children's Hospital, Hefei, China

**Keywords:** children, clinical outcome, Cox regression analysis, Epstein–Barr virus, length of stay, viral load

Affiliation “Department of Pediatric Nephrology, Anhui Provincial Children's Hospital, Hefei, China” was erroneously given as “Department of Nephrology, Anhui Provincial Children's Hospital, Hefei, China”.

Author Zhi Yang, Qingfeng Fang and Biquan Chen were erroneously assigned to affiliation “Department of Nephrology, Anhui Provincial Children's Hospital, Hefei, China”. This affiliation has now been removed for authors Zhi Yang, Qingfeng Fang and Biquan Chen.

Affiliation “1. Department of Pediatric Infectious Disease, Anhui Provincial Children's Hospital, Hefei, China” was omitted from the paper. This affiliation has now been added for authors Zhi Yang, Qingfeng Fang and Biquan Chen.

Affiliation “3. Department of Pediatric Nephrology, Anhui Provincial Children's Hospital, Hefei, China' was erroneously omitted for author Yuan Xu. This affiliation has now been added for author Yuan Xu.

The original version of this article has been updated.

